# Phase Angle and Handgrip Strength as Predictors of Clinical Outcomes in Hospitalized COVID-19 Patients

**DOI:** 10.3390/nu15061409

**Published:** 2023-03-15

**Authors:** Androniki Papaemmanouil, Dimitra Rafailia Bakaloudi, Konstantina Gkantali, Georgios Kalopitas, Simeon Metallidis, Georgios Germanidis, Michael Chourdakis

**Affiliations:** 1 Laboratory of Hygiene, Social & Preventive Medicine and Medical Statistics, School of Medicine, Faculty of Health Sciences, Aristotle University of Thessaloniki, 54124 Thessaloniki, Greece; 2 Division of Oncology, Department of Medicine, University of Washington, Seattle, WA 98109, USA; 3 Division of Infectious Diseases, 1st Department of Internal Medicine, AHEPA University Hospital, School of Medicine, Faculty of Health Sciences, Aristotle University of Thessaloniki, 54636 Thessaloniki, Greece; 4 Division of Gastroenterology and Hepatology, 1st Department of Internal Medicine, AHEPA University Hospital, School of Medicine, Faculty of Health Sciences, Aristotle University of Thessaloniki, 54636 Thessaloniki, Greece

**Keywords:** COVID-19, handgrip strength, phase angle, SARS-CoV-2 virus

## Abstract

Phase angle (PhA) and muscle strength are predictors of clinical outcomes in critically ill patients. Malnutrition may affect body composition measurements. The aim of this prospective study was to investigate the association between PhA and handgrip strength (HGS), and clinical outcomes in hospitalized COVID-19 patients. The study included a total of 102 patients. Both PhA and HGS were measured twice, within 48 h of hospital admission and on the 7th day of hospitalization. The primary outcome was the clinical status on the 28th day of hospitalization. Secondary outcomes included the hospital length of stay (LOS), the concentrations of ferritin, C-reactive protein and albumin, oxygen requirements and the severity of pneumonia. A one-way analysis of variance (ANOVA) test and Spearman r_S_ correlation coefficient were used for statistical analysis. No differences were found for PhA [on day 1 (*p* = 0.769) and day 7 (*p* = 0.807)] and the primary outcome. A difference was found between HGS on day 1 and the primary outcome (*p* = 0.008), while no difference was found for HGS on day 7 (*p* = 0.476). Body mass index was found to be associated with the oxygen requirement on day 7 (*p* = 0.005). LOS was correlated neither with PhA (r_s_ = −0.081, *p* = 0.422) nor with HGS (r_s_ = 0.137, *p* = 0.177) on the first day. HGS could be a useful indicator of clinical outcomes in COVID-19 patients, while PhA does not seem to have a clinical impact. However, further research is needed to validate the results of our study.

## 1. Introduction

A newly discovered coronavirus disease was revealed in Wuhan, China, in late 2019 (COVID-19) [1], caused by the SARS-CoV-2 virus. In January 2020, the World Health Organization (WHO) Emergency Committee declared COVID-19 as an international health crisis [2] due to the growing incidence of a severe acute respiratory syndrome [3,4] leading to pneumonia [5]. COVID-19 has been associated with considerable lung abnormalities in computed tomography (CT) scans [6], and high rates of hospitalization [7], where life-support interventions including mechanical ventilation are often needed [8].

According to WHO statistics, 630,832,131 confirmed cases of COVID-19 had been reported until 11 November 2022, including 6,584,104 deaths globally [9]. Risk factors of severe disease and mortality among patients hospitalized with COVID-19 are old age, male sex, current chronic kidney disease (CKD), cardiovascular diseases (CVD), chronic obstructive pulmonary disease (COPD), hypertension, diabetes, cancer, obesity and immunodeficiencies [10,11,12,13]. It has been identified that patients with COVID-19 who suffer from the aforementioned comorbidities have a higher risk of malnutrition and subsequently a further risk factor for worse outcomes [14].

Among the clinical symptoms, fever, dry cough, dyspnea, headache, fatigue, muscle weakness and myalgia are reported [15]. COVID-19 patients may also suffer from decreased appetite, decreased and altered taste, early saturation, decreased smell, nausea, vomiting and diarrhea [16]. These clinical manifestations could result in failure to meet nutritional needs and, as a consequence, result in weight and muscle loss and malnutrition [16]. Furthermore, the need for invasive and non-invasive ventilation makes the delivery of the patient’s nutritional needs much more difficult. Malnutrition-related causes in COVID-19 patients also include reduced mobility, disease-related catabolic procedures, inflammation and sepsis [14]. Generally, in-hospital malnutrition is associated with prolonged hospital stay, high treatment costs, poor disease recovery, and increased morbidity and mortality [17]. Progressive skeletal muscle loss and muscle strength contributes to the manifestation of sarcopenia [18].

Common laboratory abnormalities in COVID-19 patients include leukopenia, thrombocytopenia, increased levels of inflammatory markers such as C-reactive protein (CRP) and cardiac biomarkers, decreased albumin levels, and deviated serum markers of renal and liver function [19]. Albumin, in particular, which is a marker of patients’ nutritional status, has been associated with COVID-19 severity [20].

Moreover, impaired nutritional status, body composition and bioelectrical impedance parameters have been associated with an increased risk of death in various pathological conditions [21], and according to previous studies, phase angle (PhA) seems to be decreased in COVID-19 patients [22]. PhA refers to the angular shift (phase difference) between voltage and current sinusoidal waveforms [23]. PhA reflects membrane integrity, cell mass and hydration status [11]. Evidence suggests that decreased PhA is associated with higher rates of mortality, mechanical ventilation needs and comorbidities, as well as prolonged hospital stay in COVID-19 patients [11,22]. Additionally, low PhA is related to COVID-19 severity [24], as it has been further inversely associated with the length of intensive care unit (ICU) stay and the duration of mechanical ventilation [11]. Thus, PhA has been suggested as a prognostic factor among hospitalized COVID-19 patients [22].

As in most severe infections, muscle mass seems to be negatively affected in COVID-19 patients [25]. At the same time evidence suggests that limited muscle function can be associated with a higher risk of severe COVID-19 disease [26]. Moreover, results from a recent study indicated that handgrip strength (HGS) can be used as a risk predictor of future short-term adverse clinical events, such as endotracheal intubation for COVID-19 patients [27].

It is believed that body composition and muscle strength measurements could also be useful as predictors of clinical outcomes in hospitalized COVID-19 patients [21,22,25,26,27], guiding individualized therapeutic approaches. Therefore, in this study, we aimed to observe the potential relationship between PhA and HGS, as well as other anthropometric and biochemical parameters, and the clinical outcomes of hospitalized COVID-19 patients.

## 2. Materials and Methods

### 2.1. Study Population

This study was designed as an observational study which has been conducted according to the Declaration of Helsinki criteria [28], and after the approval of the hospital’s ethics committee (48/01.11.2021). Written informed consent was obtained from all eligible patients. Eligible patients were individuals from 18 to 75 years old admitted to “AHEPA” General Hospital of Thessaloniki, Greece, with a PCR-confirmed SARS-CoV-2 infection.

### 2.2. Exclusion Criteria

Exclusion criteria included individuals <18 or >75 years old, pregnant women, individuals with liver cirrhosis, on dialysis, with end-stage cancer disease or with any severe physical disability. Patients suitable for outpatient treatment as well as those directly admitted to the ICU were also excluded.

### 2.3. Demographics, Comorbidities and Laboratory Values

All clinical data were collected in the course of routine clinical procedures. Collected demographic information included patients’ age, sex, SARS-CoV-2 vaccination status, smoking status, household contact, and the presence of underlying diseases, including hypertension, diabetes mellitus, CVD, dyslipidemia, obesity (BMI ≥ 35 kg/m^2^), COPD, cancer and autoimmune diseases. Body weight and height were also self-reported in order to calculate body mass index (BMI). BMI was calculated as measured weight divided by height squared. Laboratory data that were examined were serum albumin, CRP, Interleukin-6 (IL-6), neutrophil to lymphocyte ratio, ferritin, uric acid, phosphorus and d-dimers.

### 2.4. Primary and Secondary Outcomes

The study endpoint of measurement of clinical outcome was the patient’s clinical status after 28 days. After the 28-day timeframe, the clinical status of patients was recorded as “hospital discharge”, “hospitalization” or “death”. Length of hospital stay (LOS) was calculated from the admission to the Infectious Diseases Department to the discharge from the same service, expressed in days. Mortality was defined as in-hospital death occurring during hospitalization due to COVID-19 infection. The primary outcome of this study was the examination of the 28-day clinical status. Secondary outcomes included the evaluation of hospitalization length, BMI, CRP, ferritin and albumin concentrations, oxygen requirements and stages of pneumonia.

### 2.5. Body Composition Measurements

Our protocol included two measurements of bioelectrical impedance analysis (BIA) and HGS for each patient. The first measurement was performed by trained staff on the day of patient’s admission to the hospital, as in previous studies [29]. The second one was performed on the 7th day of hospitalization (follow-up measurement). Patients requiring ICU admission and/or mechanical ventilation and patients discharged before the 7th day of hospitalization were not part of the follow-up measurements and were finally excluded from our study.

#### 2.5.1. Phase Angle

BIA is a validated, non-invasive method for assessing body composition [30]. PhA is a BIA-derived parameter that represents an indicator of cellular health [11]. This parameter is calculated by the ratio between reactance (Xc) and resistance (R) [11]. Although the typical range varies with sex and age, a PhA value greater than “six” is generally regarded as healthy [22]. A PhA of < 4.8° has been considered as an independent predictor of mortality in the ICU [31]. 

In this study, BIA was performed with the patient in a supine position using a multifrequency portable device (Bodystat Quadscan 4000), according to manufacturer’s manual instructions. PhA (°) and other BIA-derived parameters were also recorded.

#### 2.5.2. Handgrip Strength

HGS is a straightforward, repeatable, and affordable method for assessing muscular strength in clinical practice [26]. It is also used for the diagnose of sarcopenia [32] and it is associated with several chronic diseases, LOS and mortality [33]. It involves using a dynamometer to determine the maximal static force that a hand can exert [26]. Low HGS is defined as <27 kg for men and <16 kg for women [34].

In this study, HGS was measured through a calibrated Jamar hand dynamometer. Values of HGS were obtained in kilograms (kg). Three measurements were taken from each patient on each visit, of which the largest measurement was recorded. The measurement was performed in a sitting position, with the patients’ elbow at a 90-degree angle. 

### 2.6. Assessment of Severity of COVID-19

Regarding the severity of the pneumonia, it was classified by its radiological severity and the patient’s oxygen requirements. Regarding its radiological severity, it was classified from stage 1 (mild) to stage 5 (severe) after performing a chest CT scan. With regard to the severity of the respiratory failure, oxygen support needs were recorded as follows: (1) on air—without oxygen support, (2) up to 4 L of oxygen support per day, (3) >4 L of oxygen support per day to venturi 50% mask (Fracture of inspired oxygen (FiO2:0.5)), (4) non-invasive ventilation (high flow nasal cannula or face mask), (5) intubation.

### 2.7. Statistical Analysis

Descriptive statistics were used to summarize the baseline characteristics of the included patients. Continuous variables were expressed as mean (SD) and median (Q1, Q3). Categorical variables were expressed as absolute values and percentages. Statistical tests which were used for nonparametric variables were Wilcoxon signed rank test and Kruskal–Wallis test. The one-way analysis of variance (ANOVA) test was performed using the Bonferroni method, for normal contributed variables. Furthermore, the Spearman r_S_ correlation coefficient was used to examine the correlation between quantitative variables. Statistical tests were considered significant if *p* was < 0.05.

## 3. Results

### 3.1. Patients Characteristics

A total of 102 patients (median age 59 years, median BMI 29, 83.3% non-smokers) were enrolled in the study from November 2021 to March 2022. A total of 59.8% of patients were males. Details on the baseline characteristics of enrolled patients can be found in Table 1.

Clinical characteristics between the day of admission (first day) and the seventh day of hospitalization can be seen in Table 2. The median PhA was 6.1° (5.2–8.3) and 5.95° (4.8–9.8) for days 1 and 7, respectively. The mean HGS on the first day was 34.1 (14.1) kg and at the seventh day was 34.9 (14.5) kg. The median PhA and mean HGS values for males and females can be found in the aforementioned table. The mean concentration of CRP was 7.6 (5.6) mg/dL (1st day) and 1.3 (1.6) mg/dL (7th day). On the first day, the mean concentration of albumin and median concentration of ferritin were 3.6 (0.4) g/dL and 577 (46–986) ng/mL, respectively. PhA between the first and the seventh day did not present any difference (*p* = 0.540). With regard to oxygen requirements, on the first day, the majority among our group did not require oxygen support (22.5%) or received ≤4 L O_2_ (33.3%)_,_ whereas none of the patients was intubated; on the seventh day, 10.8% of the patients were intubated. By the 28th day, 84 patients had been discharged from hospital, four patients were still hospitalized and 12 patients were deceased.

### 3.2. Primary Outcomes

When it comes to the primary outcome, there was no difference between the PhA of both the first (*p* = 0.769) and the seventh (*p* = 0.807) day and the clinical status on the 28th day. Following the same pattern, no difference was found between the HGS of the 7th (*p* = 0.476) day and the clinical status on the 28th day. However, a difference was found between the HGS of the 1st day and the clinical status on the 28th day (*p* = 0.008). Further multiple comparisons showed that individuals who were discharged from hospital had greater mean HGS on the first day (40.1 kg) compared to those individuals who died (30.9 kg) (*p* = 0.04). The mean HGS (Day 1) in patients who were hospitalized at the 28th day was not differed than the HGS of individuals who were discharged from hospital or died (*p* = 0.972). Primary outcome comparisons are shown in Table 3.

### 3.3. Secondary Outcomes

When it comes to secondary outcomes, LOS was correlated neither with the PhA (Day 1, r_s_ = −0.081, *p* = 0.422), nor with the HGS (Day 1, r_s_ = 0.137, *p* = 0.177). Moreover, PhA on the first day of measurement did not correlate with the concentrations of ferritin (r_s_ = 0.092, *p* = 0.370) or CRP (r_s_ = −0.043, *p* = 0.680) upon hospital admission. However, PhA obtained from the first day of measurement correlated positively with the concentration of albumin on the first day (r_s_ = 0.286, *p* = 0.006). In addition, the distribution of PhA on the first day was the same across the different categories of oxygen requirements (Day 1, *p* = 0.733 and Day 7, *p* = 0.603), as well as across the categories of the stages of pneumonia (*p* = 0.371). On the seventh day of hospitalization, a difference was found regarding oxygen requirements and BMI (*p* = 0.005). The median BMI of patients that did not require oxygen support was lower than of patients who needed up to 4 L of oxygen support per day (*p* = 0.002). Secondary outcome correlations are shown in Table 4.

## 4. Discussion

In this study, we examined the potential relationship between PhA, HGS and the clinical outcomes of hospitalized COVID-19 patients. The results of this study showed no difference between PhA (neither for Day 1 nor for Day 7) and the clinical status on the 28th day. Similarly, no difference was found between PhA (Day 1) and LOS, nor between PhA (Day 1) and either ferritin or CRP concentrations. A difference was found between PhA (Day 1) and albumin concentration. The HGS of the first day was correlated with the clinical status of the 28th day of the patient. However, no difference was found between HGS of the 7th day and the clinical status of the 28th day. Our study’s results also indicated that patients who did not require oxygen support had a lower BMI than patients who needed up to 4 L of oxygen support per day.

Recently, Del Giorno et al. found that low PhA was associated with prolonged LOS and increased mortality in COVID-19 patients [35]. A similar study from Osuna-Padilla et al. indicated that low PhA in COVID-19 patients was associated with increased 60-day mortality [21]. In addition, Cornejo-Pareha et al. found that a low PhA was associated with increased mortality at 90 days [22]. Moonen et al. also showed that PhA was inversely associated with LOS [30]. Moreover, Rosas-Carrasco et al. demonstrated that a low PhA represents a higher risk of mortality in hospitalized COVID-19 patients [36]. COVID-19 pneumonia is an inflammatory situation which promotes a greater resistance to the movement of electrical current through human tissues, so it has been associated with mortality prediction in similar studies [36]. However, the results of our study do not confirm this finding. A possible explanation of our findings contradicting the findings of previous studies could be the fact that most patients in our study had close to normal baseline PhA values at the time of admission and most of our patients were discharged from the hospital before the seventh day of hospitalization.

Moreover, in our study, no difference was found for PhA between the first and the seventh day. In a recent study, Kellnar et al. found a decrease in PhA angle measurement between the first and third day of hospitalization, with a slow regression towards hospital discharge [37]. The difference between this result and the result of our study can be due to the longer time interval until the second measurement. However, we believe that a 7-day time interval between consecutive measurement of PhA can be more representative for the body composition changes that might occur, than a three-day interval.

In this study, no differences were identified between PhA (Day 1) and the concentration of ferritin or CRP at the time of hospital admission. Our study is the first one that examined the correlation between PhA and ferritin and CRP concentrations in patients with COVID-19. CRP has been shown to be associated with the progression of COVID-19 [38]. Inflammation may well affect body fluid status and therefore PhA measurements as well [22]. However, correlations between PhA, CRP and ferritin concentrations were not found, possibly due to the fact that a portion of the patients in our study were admitted to the hospital after the first few days of the acute inflammatory state. 

Moreover, in our study, PhA was found to be positively correlated with albumin concentration. Inflammatory mediators reduce albumin synthesis and increase albumin catabolism when a patient is critically ill in favor of other acute phase reactants [39]. According to previous studies, lower serum albumin concentrations are associated with the disease severity and adverse outcomes in COVID-19 patients [40], as well as with poor prognosis [41]. PhA and albumin are both nutritional status indicators, but the presence of inflammation in hospitalized patients may affect their values and correlations [22]. Further research is required in this field to better determine and explain this correlation. 

The distribution of PhA (Day 1) was similar across all categories of oxygen requirements (for both Days 1 and 7) and across the different categories of the stages of pneumonia. This result could also be explained by the fact that PhA measurements were close to normal in most of our population at the time of hospital admission.

Furthermore, our study indicated a difference between HGS of the 1st day and the clinical status on the 28th day. HGS of the day of admission to the hospital was found to be lower in individuals who deceased compared to the HGS of individuals that were finally discharged, meaning that such a measurement could be used as a prognostic predictor. 

No difference was found between HGS of the 7th day and the clinical status on the 28th day, nor between HGS of the 1st day and LOS. According to previous studies, lower HGS was associated with higher mortality from a respiratory disease [42]. Cheval et al. indicated that muscle strength is an independent risk factor for COVID-19 severity in adults aged 50 and older [27]. In addition, Kara et al. [43] found that low HGS can be a predictor of severe COVID-19 disease.

Muscle loss could result from prolonged immobility, the use of antiviral drugs and undernutrition [25]. COVID-19, as a hypercatabolic condition, is also related to enhanced proteolysis, due to severe inflammatory stress [44]. Muscular strength has also been identified as a risk factor for all-cause mortality [45]. In addition, Gil et al. found that low muscle mass could be associated with a higher LOS in COVID-19 patients [46]. Similarly, negative correlations were found between HGS and LOS in the study performed by Sevilla et al. [47]. In the study of Pucci and colleagues, HGS was found to predict risk of endotracheal intubation [24].

This study also indicated that median BMI was lower in patients that did not require oxygen support than among patients who needed up to 4 L of oxygen support per day. Several other studies have demonstrated that overweight and obesity have been associated with mortality and increased disease severity [48]. The mechanical effects of obesity can cause airway narrowing and increased resistance. Airway narrowing in obesity is associated with airway closure and airway hyperresponsiveness [49].

Increased dietary intake is a major factor leading to obesity and, especially during the period of the COVID-19 lockdown, both body weight and BMI were increased [50]. Obesity appears to be associated with a greater likelihood of respiratory complications and mechanical ventilation in COVID-19 patients [49]. In the study of Palaiodimos et al., a BMI ≥ 35 kg/m^2^ was found to be associated with increased oxygen requirements [51]. A meta-analysis including 4752 hospitalized overweight/obese patients with COVID-19 showed that those with overweight were more likely to require oxygen support [52]. A systematic review from Soeroto et al. concluded that BMI is an important routine measurement that should be evaluated in COVID-19 patients, because of its relationship with the disease’s outcome [49].

Body composition may be affected by the patients’ nutritional status. COVID-19 worsens patients’ nutritional status, even before hospital admission [16]. Malnutrition has been demonstrated as a risk factor for severity and mortality from virus-related pneumonia, as well as for hospitalization in residents with home-acquired pneumonia [38]. COVID-19 infection causes an inflammatory and catabolic condition, while oxygen support makes the feeding procedure difficult [53]. As a result, COVID-19 patients are vulnerable to malnutrition [53]. For COVID-19 patients in particular, LOS and ICU stay, as well as mortality, seems to be increased in malnourished patients in comparison to well-nourished patients [44]. In the study of Bedock et al., 42.1% of COVID-19 patients were malnourished, while 18.4% of them were severely malnourished [38].

It is hypothesized that PhA could reflect nutritional status, as it represents characteristics of human tissues [54]. However, a systematic review using the Grading of Recommendations, Assessment, Development, and Evaluation (GRADE) approach from Rinaldi et al. [54], showed that there is not enough evidence to consider PhA as an accurate indicator of malnutrition. On the other hand, the study from Jansen et al. [29] in critically ill patients showed that a decreased standardized PhA (sPhA) increased the possibility for a patient to be diagnosed with malnutrition. Previous studies have also evaluated other nutritional markers, such as HGS as predictors of malnutrition [55].

Nutritional status and swallowing function are also related, mainly when muscle loss is present [29]. The study from Reyes-Torres et al. indicated a high prevalence of dysphagia in COVID-19 patients, which was explained by the implementation of mechanical ventilation [29]. Another muscle-related dysfunction is sarcopenia, which may play a relevant role in COVID-19 outcome [54]. Xu et al. demonstrated that the prevalence of sarcopenia was 48% based on evidence from 5407 COVID-19 patients. Sarcopenia in COVID-19 patients has been recently reported to be associated with increased complications and mortality [55]. As HGS is a marker of sarcopenia [24], it is suggested to be used as a prognostic marker of COVID-19 patients [54] and patients with low HGS on admission should be closely monitored as patients at greater risk of adverse outcomes.

The ability to collect a variety of data, such as body composition variables (PhA, BMI, HGS) and laboratory data (albumin, ferritin, CRP), and the daily monitoring of patients by clinicians, are included in the strengths of our study. A strong advantage of our study is that we managed to conduct a follow-up with patients and make serial measurements of the outcomes of interest. Moreover, the fact that the clinical doctors who monitored the patients were different from the researchers who collected and processed the data, meant that they were blinded to study results, contributing to reducing biases, and this is among our study’s strengths. 

On the other hand, our rather small sample size and the fact the data were obtained from a single hospital center may impair the generalization of our conclusions. Furthermore, the variety in disease stages during the measurement period, and possible human errors during measurements are included in the limitations of our study. Another weakness of our study is the fact that we did not manage to assess the nutritional status with a validated screening tool or the presence of dysphagia and other nutrition-related symptoms, which may affect PhA and HGS.

As HGS reflects patients’ muscle strength, it could be a useful routine measurement that should be used in hospitalized COVID-19 patients because of its relationship with the disease’s outcomes. Further studies with a bigger sample size are required for the confirmation of this correlation. More studies are needed to elucidate the predictive discretion of BIA parameters, such as PhA, in COVID-19 patients.

## 5. Conclusions

Our study suggests that HGS at hospital admission could be a prognostic predictor in COVID-19 patients. The relationship between PhA and the clinical features of COVID-19 patients remains unclear, so further research is needed in order to better determine the role of this indicator in predicting severity and outcome in COVID-19 patients.

## Figures and Tables

**Table 1:** Baseline characteristics of hospitalized COVID-19 patients.

	*N* (%)	
Age (years)	59 (47.5–67) *	
BMI (kg/m^2^)	29 (25–32.5)	
Sex	Male 61 (59.8%) ^#^	Female 41 (40.2%)
	Yes	No
Smokers	17 (16.7%)	85 (83.3%)
Hypertension	31 (30.4%)	71 (69.6%)
Diabetes	16 (15.7%)	86 (84.3%)
Cardiovascular Disease	9 (8.8%)	93 (91.2%)
Dyslipidemia	26 (25.5%)	76 (74.5%)
Obesity	16 (15.7%)	86 (84.3%)
Cancer	8 (7.8%)	94 (92.2%)
Autoimmune Disease	9 (8.8%)	93 (91.2%)
Pulmonary Disease	5 (4.9%)	97 (95.1%)

* median (Q1, Q3), ^#^ Frequency (% of total).

**Table 2:** Clinical characteristics of COVID-19 patients for Days 1 and 7.

		Day 1	Day 7
Phase Angle (°)	Total	6.1 (5.2–8.3)	5.95 (4.8–9.8)
	Male	6.5 (5.4–8.6)	6.25 (4.9–10.7)
	Female	5.8 (4.9–8.2)	5.4 (4.9–9.7)
Lean Body Mass (kg)		57.3 (13.4)	57.5 (14.0)
Dry Lean Body Mass (kg)		14.5 (5.7)	14.5 (5.5)
Fat Mass (kg)		39.4 (18.8)	38.6 (17.6)
Handgrip Strength (kg)	Total	34.1 (14.1)	34.9 (14.5)
	Male	39.9 (11.8)	40.9 (12.2)
	Female	22 (6.2)	23.1 (5)
Arm circumference (cm)		36.4 (5.2)	35.5 (5.2)
CRP (mg/dL)		7.6 (5.6)	1.3 (1.6)
Albumin (g/dL)		3.6 (0.4)	-
IL-6 (pm/mL)		32.5 (17.8–63.1)	-
NET/LYMA (k/μL)		3.5 (2.3–5.6)	3.6 (2.4–5.8)
Ferritin (ng/mL)		577 (246–986)	-
Uric acid (mg/dL)		4.5 (3.4–5.9)	-
Phosphorus (mg/dL)		3.9 (0.7)	-
D-dimers (ng/mL)		227 (145–387)	243 (161–353)
Oxygen requirements *N* (%)			
Without support		23 (22.5%)	51 (50.0%)
≤4 L O_2_		34 (33.3%)	15 (14.7%)
>4 L		27 (26.5%)	12 (11.8%)
High flow		18 (17.6%)	11 (10.8%)
Intubation		-	11 (10.8%)

Variables are presented as mean (SD), median (Q1, Q3), SD: Standard deviation.

**Table 3:** Primary outcome comparisons.

	28-Day Outcome
PhA (Day 1)	*p* = 0.769
PhA (Day 7)	*p* = 0.807
HGS (Day 1)	*p* = 0.008
HGS (Day 7)	*p* = 0.476

**Table 4:** Secondary outcome correlations.

	LOS		Ferritin (ng/dL)		CRP (mg/dL)		Albumin (mg/dL)	
	r_s_	*p*	r_s_	*p*	r_s_	*p*	r_s_	*p*
PhA (Day 1)	−0.081	0.422	0.092	0.370	−0.043	0.680	0.286	0.006
HGS (Day 1)	0.137	0.177	-	-	-	-	-	-

r_s_: Spearman correlation coefficient. *p*: *p*-value.

## Data Availability

Not applicable.

## References

[1] Grant W.B., Lahore H., McDonnell S.L., Baggerly C.A., French C.B., Aliano J.L., Bhattoa H.P. (2020). Evidence that Vitamin D Supplementation Could Reduce Risk of Influenza and COVID-19 Infections and Deaths. Nutrients.

[2] Velavan T.P., Meyer C.G. (2020). The COVID-19 Epidemic. Trop. Med. Int. Health.

[3] Attaway A.H., Scheraga R.G., Bhimraj A., Biehl M., Hatipoğlu U. (2021). Severe covid-19 pneumonia: Pathogenesis and clinical management. BMJ.

[4] Cronin J.N., Camporota L., Formenti F. (2021). Mechanical ventilation in COVID-19: A physiological perspective. Exp. Physiol..

[5] Lai C.C., Shih T.P., Ko W.C., Tang H.J., Hsueh P.R. (2020). Severe Acute Respiratory Syndrome Coronavirus 2 (SARS-CoV-2) and Coronavirus Disease-2019 (COVID-19): The Epidemic and the Challenges. Int. J. Antimicrob. Agents.

[6] Van der Sar-van der Brugge S., Talman S., Winter L.B.-D., de Mol M., Hoefman E., van Etten R.W., De Backer I.C. (2021). Pulmonary function and health-related quality of life after COVID-19 pneumonia. Respir. Med..

[7] Baccolini V., Migliara G., Isonne C., Dorelli B., Barone L.C., Giannini D., Marotta D., Marte M., Mazzalai E., Alessandri F. (2021). The impact of the COVID-19 pandemic on healthcare-associated infections in intensive care unit patients: A retrospective cohort study. Antimicrob. Resist. Infect. Control..

[8] Brioni M., Meli A., Grasselli G. (2022). Mechanical Ventilation for COVID-19 Patients. Semin. Respir. Crit. Care Med..

[9] World Health Organization Coronavirus Disease (COVID-19) Pandemic. https://www.who.int/europe/emergencies/situations/covid-19.

[10] Hergens M.-P., Bell M., Haglund P., Sundström J., Lampa E., Nederby-Öhd J., Östlund M.R., Cars T. (2022). Risk factors for COVID-19-related death, hospitalization and intensive care: A population-wide study of all inhabitants in Stockholm. Eur. J. Epidemiol..

[11] Alves E.A.S., Salazar T.C.D.N., Silvino V.O., Cardoso G.A., dos Santos M.A.P. (2022). Association between phase angle and adverse clinical outcomes in hospitalized patients with COVID-19: A systematic review. Nutr. Clin. Pr..

[12] Bunnell K.M., Thaweethai T., Buckless C., Shinnick D.J., Torriani M., Foulkes A.S., Bredella M.A. (2021). Body composition predictors of outcome in patients with COVID-19. Int. J. Obes..

[13] Bhaskaran K., Rentsch C.T., Hickman G., Hulme W.J., Schultze A., Curtis H.J., Wing K., Warren-Gash C., Tomlinson L., Bates C.J. (2022). Overall and cause-specific hospitalisation and death after COVID-19 hospitalisation in England: A cohort study using linked primary care, secondary care, and death registration data in the OpenSAFELY platform. PLoS Med..

[14] Barazzoni R., Bischoff S.C., Breda J., Wickramasinghe K., Krznaric Z., Nitzan D., Pirlich M., Singer P. (2020). ESPEN expert statements and practical guidance for nutritional management of individuals with SARS-CoV-2 infection. Clin. Nutr..

[15] Mesquita R.D.R., Junior L.C.F.S., Santana F.M.S., de Oliveira T.F., Alcântara R.C., Arnozo G.M., Filho E.R.D.S., dos Santos A.G.G., da Cunha E.J.O., de Aquino S.H.S. (2020). Clinical manifestations of COVID-19 in the general population: Systematic review. Wien. Klin. Wochenschr..

[16] Wierdsma N.J., Kruizenga H.M., Konings L.A., Krebbers D., Jorissen J.R., Joosten M.-H.I., van Aken L.H., Tan F.M., van Bodegraven A.A., Soeters M.R. (2021). Poor nutritional status, risk of sarcopenia and nutrition related complaints are prevalent in COVID-19 patients during and after hospital admission. Clin. Nutr. ESPEN.

[17] Norman K., Pichard C., Lochs H., Pirlich M. (2008). Prognostic impact of disease-related malnutrition. Clin. Nutr..

[18] Evans W.J. (2010). Skeletal muscle loss: Cachexia, sarcopenia, and inactivity. Am. J. Clin. Nutr..

[19] Esakandari H., Nabi-Afjadi M., Fakkari-Afjadi J., Farahmandian N., Miresmaeili S.M., Bahreini E. (2020). A Comprehen-sive Review of COVID-19 Characteristics. Biol. Proced. Online.

[20] Li Y., Zhu C., Zhang B., Liu L., Ji F., Zhao Y., Cheng J., Shao H., Guan X., Ming F. (2021). Nutritional status is closely related to the severity of COVID-19: A multi-center retrospective study. J. Infect. Dev. Ctries..

[21] Osuna-Padilla I.A., Rodríguez-Moguel N.C., Rodríguez-Llamazares S., Aguilar-Vargas A., Casas-Aparicio G.A., Ríos-Ayala M.A., Hernández-Cardenas C.M. (2021). Low phase angle is associated with 60-day mortality in critically ill patients with COVID-19. J. Parenter. Enter. Nutr..

[22] Cornejo-Pareja I., Vegas-Aguilar I.M., García-Almeida J.M., Bellido-Guerrero D., Talluri A., Lukaski H., Tinahones F.J. (2021). Phase angle and standardized phase angle from bioelectrical impedance measurements as a prognostic factor for mortality at 90 days in patients with COVID-19: A longitudinal cohort study. Clin. Nutr..

[23] Di Vincenzo O., Marra M., Di Gregorio A., Pasanisi F., Scalfi L. (2020). Bioelectrical impedance analysis (BIA)-derived phase angle in sarcopenia: A systematic review. Clin. Nutr..

[24] Moonen H.P.F.X., van Zanten F.J.L., Driessen L., de Smet V., Slingerland-Boot R., Mensink M., van Zanten A.R.H. (2020). Association of bioelectric impedance analysis body composition and disease severity in COVID-19 hospital ward and ICU patients: The BIAC-19 study. Clin. Nutr..

[25] Ali A.M., Kunugi H. (2021). Skeletal Muscle Damage in COVID-19: A Call for Action. Medicina.

[26] Pucci G., D’Abbondanza M., Curcio R., Alcidi R., Campanella T., Chiatti L., Gandolfo V., Veca V., Casarola G., Leone M.C. (2022). Handgrip strength is associated with adverse outcomes in patients hospitalized for COVID-19-associated pneumonia. Intern. Emerg. Med..

[27] Cheval B., Sieber S., Maltagliati S., Millet G.P., Formánek T., Chalabaev A., Cullati S., Boisgontier M.P. (2021). Muscle strength is associated with COVID-19 hospitalization in adults 50 years of age or older. J. Cachex Sarcopenia Muscle.

[28] World Medical Association (2013). World Medical Association Declaration of Helsinki: Ethical principles for medical research involving human subjects. JAMA.

[29] Jansen A.K., Gattermann T., Fink J.D.S., Saldanha M.F., Rocha C.D.N., Moreira T.H.D.S., Silva F.M. (2019). Low standardized phase angle predicts prolonged hospitalization in critically ill patients. Clin. Nutr. ESPEN.

[30] Moonen H.P., Bos A.E., Hermans A.J., Stikkelman E., van Zanten F.J., van Zanten A.R. (2021). Bioelectric impedance body composition and phase angle in relation to 90-day adverse outcome in hospitalized COVID-19 ward and ICU patients: The prospective BIAC-19 study. Clin. Nutr. ESPEN.

[31] Reyes-Torres C.A., Flores-López A., Osuna-Padilla I.A., Hernández-Cárdenas C.M., Serralde-Zúñiga A.E. (2021). Phase angle and overhydration are associated with post-extubating dysphagia in patients with COVID-19 discharged from the ICU. Nutr. Clin. Pract..

[32] Lee S.Y. (2021). Handgrip Strength: An Irreplaceable Indicator of Muscle Function. Ann. Rehabil. Med..

[33] Lee S.H., Gong H.S. (2020). Measurement and Interpretation of Handgrip Strength for Research on Sarcopenia and Osteoporosis. J. Bone Metab..

[34] Wiśniowska-Szurlej A., Ćwirlej-Sozańska A., Kilian J., Wołoszyn N., Sozański B., Wilmowska-Pietruszyńska A. (2021). Reference values and factors associated with hand grip strength among older adults living in southeastern Poland. Sci. Rep..

[35] Del Giorno R., Quarenghi M., Stefanelli K., Rigamonti A., Stanglini C., De Vecchi V., Gabutti L. (2020). Phase angle is associated with length of hospital stay, readmissions, mortality, and falls in patients hospitalized in internal-medicine wards: A retrospective cohort study. Nutrition.

[36] Rosas-Carrasco O., Núñez-Fritsche G., López-Teros M.T., Acosta-Méndez P., Cruz-Oñate J.C., Navarrete-Cendejas A.Y., Delgado-Moreno G. (2022). Low muscle strength and low phase angle predicts greater risk to mortality than severity scales (APACHE, SOFA, and CURB-65) in adults hospitalized for SARS-CoV-2 pneumonia. Front. Nutr..

[37] Kellnar A., Hoppe J.M., Brunner S., Stremmel C. (2021). Hospitalization for COVID-19 is associated with significant changes in body composition. Clin. Nutr. ESPEN.

[38] Bedock D., Lassen P.B., Mathian A., Moreau P., Couffignal J., Ciangura C., Poitou-Bernert C., Jeannin A.-C., Mosbah H., Fadlallah J. (2020). Prevalence and severity of malnutrition in hospitalized COVID-19 patients. Clin. Nutr. ESPEN.

[39] Rabbani G., Ahn S.N. (2021). Review: Roles of human serum albumin in prediction, diagnoses and treatment of COVID-19. Int. J. Biol. Macromol..

[40] Paliogiannis P., Mangoni A.A., Cangemi M., Fois A.G., Carru C., Zinellu A. (2021). Serum albumin concentrations are associated with disease severity and outcomes in coronavirus 19 disease (COVID-19): A systematic review and meta-analysis. Clin. Exp. Med..

[41] Zerbato V., Sanson G., De Luca M., Di Bella S., di Masi A., Caironi P., Marini B., Ippodrino R., Luzzati R. (2022). The Impact of Serum Albumin Levels on COVID-19 Mortality. Infect. Dis. Rep..

[42] Mey R., Calatayud J., Casaña J., Torres-Castro R., Cuenca-Martínez F., Suso-Martí L., Andersen L., López-Bueno R. (2022). Handgrip strength and respiratory disease mortality: Longitudinal analyses from SHARE. Pulmonology.

[43] Kara Ö., Kara M., Akın M.E., Özçakar L. (2021). Grip strength as a predictor of disease severity in hospitalized COVID-19 patients. Hear. Lung.

[44] Nicolau J., Ayala L., Sanchís P., Olivares J., Dotres K., Soler A.-G., Rodríguez I., Gómez L.A., Masmiquel L. (2021). Influence of nutritional status on clinical outcomes among hospitalized patients with COVID-19. Clin. Nutr. ESPEN.

[45] García-Hermoso A., Cavero-Redondo I., Ramírez-Vélez R., Ruiz J.R., Ortega F.B., Lee D.-C., Martínez-Vizcaíno V. (2018). Muscular Strength as a Predictor of All-Cause Mortality in an Apparently Healthy Population: A Systematic Review and Meta-Analysis of Data From Approximately 2 Million Men and Women. Arch. Phys. Med. Rehabilit..

[46] Gil S., Filho W.J., Shinjo S.K., Ferriolli E., Busse A.L., Avelino-Silva T.J., Longobardi I., de Oliveira Júnior G.N., Swinton P., Gualano B. (2021). Muscle strength and muscle mass as predictors of hospital length of stay in patients with moderate to severe COVID-19: A prospective observational study. J. Cachexia Sarcopenia Muscle.

[47] de Sevilla G.G.P., Sánchez-Pinto B. (2022). Associations between muscle strength, dyspnea and quality of life in post-COVID-19 patients. Rev. Da Assoc. Médica Bras..

[48] Silverio R., Gonçalves D.C., Andrade M.F., Seelaender M. (2021). Coronavirus Disease 2019 (COVID-19) and Nutritional Status: The Missing Link?. Adv. Nutr..

[49] Soeroto A.Y., Soetedjo N.N., Purwiga A., Santoso P., Kulsum I.D., Suryadinata H., Ferdian F. (2020). Effect of increased BMI and obesity on the outcome of COVID-19 adult patients: A systematic review and meta-analysis. Diabetes Metab. Syndr. Clin. Res. Rev..

[50] Bakaloudi D.R., Barazzoni R., Bischoff S.C., Breda J., Wickramasinghe K., Chourdakis M. (2021). Impact of the first COVID-19 lockdown on body weight: A combined systematic review and a meta-analysis. Clin. Nutr..

[51] Palaiodimos L., Kokkinidis D.G., Li W., Karamanis D., Ognibene J., Arora S., Southern W.N., Mantzoros C.S. (2020). Severe obesity, increasing age and male sex are independently associated with worse in-hospital outcomes, and higher in-hospital mortality, in a cohort of patients with COVID-19 in the Bronx, New York. Metabolism.

[52] Longmore D.K., Miller J.E., Bekkering S., Saner C., Mifsud E., Zhu Y., Saffery R., Nichol A., Colditz G., Short K.R. (2021). Diabetes and Overweight/Obesity Are Independent, Nonadditive Risk Factors for In-Hospital Severity of COVID-19: An International, Multicenter Retrospective Meta-analysis. Diabetes Care.

[53] Da Porto A., Tascini C., Peghin M., Sozio E., Colussi G., Casarsa V., Bulfone L., Graziano E., De Carlo C., Catena C. (2021). Prognostic Role of Malnutrition Diagnosed by Bioelectrical Impedance Vector Analysis in Older Adults Hospitalized with COVID-19 Pneumonia: A Prospective Study. Nutrients.

[54] Rinaldi S., Gilliland J., O’Connor C., Chesworth B., Madill J. (2018). Is phase angle an appropriate indicator of malnutrition in different disease states? A systematic review. Clin. Nutr. ESPEN.

[55] Fernández-Jiménez R., Dalla-Rovere L., García-Olivares M., Abuín-Fernández J., Sánchez-Torralvo F.J., Doulatram-Gamgaram V.K., Hernández-Sanchez A.M., García-Almeida J.M. (2022). Phase Angle and Handgrip Strength as a Predictor of Disease-Related Malnutrition in Admitted Patients: 12-Month Mortality. Nutrients.

